# Mutagen-induced phytotoxicity in maize seed germination is dependent on ROS scavenging capacity

**DOI:** 10.1038/s41598-018-32271-y

**Published:** 2018-09-19

**Authors:** Yifei Zhang, Haojie Shi, Benliang Deng

**Affiliations:** 1 Heilongjiang Provincial Key Laboratory of Modern Agricultural Cultivation and Crop Germplasm Improvement, Department of Agronomy, Heilongjiang Bayi Agricultural University, Daqing, 163319 China; 2 School of Agricultural and Food Sciences, Zhejiang Agriculture and Forest University, Hangzhou, PR China

## Abstract

Ethidium bromide (EB) and acridine orange (AO) bind to nucleic acids and are thus considered as potential mutagens. In this study, the effects of EB and AO on the germination behaviours of white, yellow, red, and purple maize seeds were investigated. The results indicate that low concentrations of EB (50 μg mL^−1^) and AO (500 μg mL^−1^) promote germination, particularly for the white and yellow seeds. However, high concentrations of EB (0.5 mg mL^−1^) and AO (5 mg mL^−1^) significantly inhibit germination, with the level of inhibition decreasing in the following order: white > yellow > red > purple. In addition, EB and AO induce H_2_O_2_ production in a concentration-dependent manner. The effects of these mutagens on seed germination were partly reversed by dimethyl thiourea, a scavenger of reactive oxygen species (ROS), and diphenylene iodonium (DPI), an inhibitor of NADPH oxidase, while the effects were enhanced by treatment with H_2_O_2_ and 3-amino-1,2,4-triazole, a specific inhibitor of catalase. In addition, AO and EB profoundly increased NADPH oxidase activities in germinating seeds. The treatment of seeds with EB and AO did not affect the growth or drought tolerance of the resultant seedlings. The findings suggest that the mechanism of mutagen toxicity is related to the induction of ROS production.

## Introduction

Ethidium bromide (EB) is an intercalating agent that is commonly used as a fluorescent tag in molecular biology for techniques such as agarose gel electrophoresis^1,2^. EB may act as a mutagen because it intercalates double-stranded DNA (i.e., EB inserts itself between the strands), deforming the DNA^3^ and potentially affecting biological processes such as DNA replication and transcription. However, the mutagenicity of EB depends on the particular organism and the circumstances of exposure^4^. EB has been shown to be mutagenic to bacteria via the Ames test, but only after treatment with liver homogenate, which simulates the metabolic breakdown of the molecule being tested^5^.

Acridines and a large number of acridine derivatives generate mutagenic action in a wide variety of organisms^6–10^. At the molecular level, acridines have been shown to cause both addition- and deletion-type frameshift mutations^6^. Acridine orange (AO) is a cell-permeable organic compound that can interact with nucleic acids via intercalation or electrostatic attraction. Thus, AO can be used as a nucleic acid-selective fluorescent cationic dye^6^. Interestingly, AO can be used in conjunction with EB to differentiate between viable, apoptotic, and necrotic cells. AO can bind with the nucleic acids in both living and dead bacteria along with other microorganisms^9^.

Reactive oxygen species (ROS) are continuously produced in plants as byproducts of aerobic metabolism^11^. These ROS can be detrimental to lipids, proteins, carbohydrates, and nucleic acids; thus, long-term exposure to ROS can accelerate plant senescence^11^. ROS are continuously produced during seed development from embryogenesis to germination and during seed storage^12–14^. As components of the cellular signalling pathway, ROS play a positive role in seed dormancy release^15^. For example, the inhibition of catalase (an enzyme for H_2_O_2_ scavenging) favours H_2_O_2_ production and seed dormancy release^16^. However, ROS over-production can arrest seed germination^13^.

Typically, germination involves the reactivation of a seed’s metabolic machinery, resulting in the emergence of the radicle and plumule^17^. In other words, during germination, the plant enters a new growth stage with a high level of mitochondrial respiration and DNA replication^18^. EB is able to bind with nucleic acids and interfere with normal mitochondrial activities^19^. However, few data are available on the effects of mutagens like EB on seed germination.

Seed germination is commonly used to evaluate the toxicity of potentially hazardous materials^20–22^. In this study, seeds of different colours (white, yellow, red, and purple) from variegated maize cob were used to evaluate the effects of EB and AO on seed germination^23^. The different coloured seeds come from the same maize cob but have different antioxidant capacities^23^. This study focused on the following key research questions: Can the nucleic acid-binding compounds EB and AO affect seed germination? What are the possible mechanisms underlying the effects of EB and AO on seed germination? Does treating seeds with EB and AO affect the subsequent seedling growth? This work is the first to report mutagen (AO and EB)-induced phytotoxicity from a ROS perspective.

## Results

### Effects of AO and EB on GR

**Figure 1:**
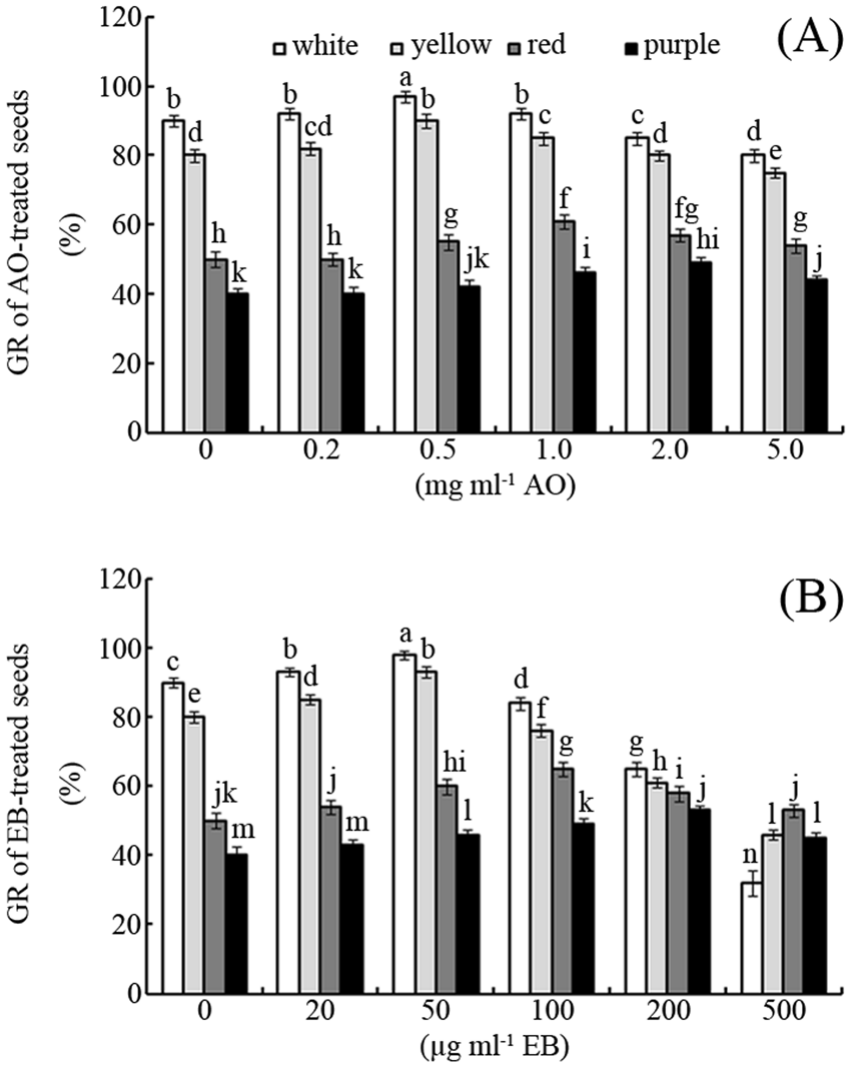
Effects of AO/EB on GR Effects of different concentrations of AO (**A**) and EB (**B**) on the seed GRs of maize. Bars represent standard deviations of means (*n* = 5); means followed by the same letter are not significantly different (*p* < 0.05) among treatments. EB, ethidium bromide; AO, acridine orange; GR, germination rate.

As shown in Fig. 1, both EB and AO significantly affected the final (day 5) GR of the maize seeds, especially the light-coloured seeds. However, the different concentrations of EB and AO had different effects on seed GR. For example, treatment with 0.5 mg mL^−1^ AO increased the GRs of the white, yellow, red, and purple seeds by approximately 8%, 13%, 10%, and 5% compared to the control, respectively (Fig. 1A; *p* < 0.05); in contrast, treatment with 5 mg mL^−1^ AO reduced these GRs by approximately 11%, 6%, 4%, and 0%, respectively (Fig. 1A; *p* < 0.05). Compared with AO, EB exhibited a greater inhibitory effect on seed GR. For example, treatment with 500 μg mL^−1^ EB reduced the final GRs of white (64%), yellow (33%) and red (4%), but enhanced that of purple seeds (5%) compared to the control, respectively (Fig. 1B; *p* < 0.05).

### Effects of AO and EB on GS

**Figure 2:**
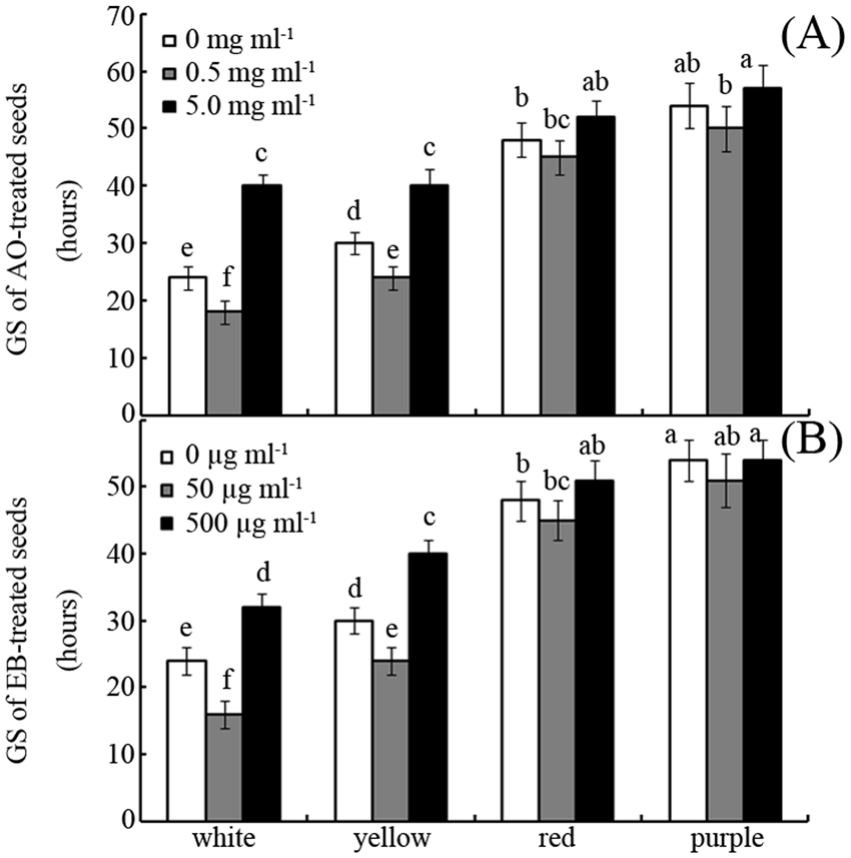
Effects of AO/EB on GS Effects of different concentrations of AO (**A**) and EB (**B**) on the GS of maize seeds. Bars represent standard deviations of means (*n* = 5); means followed by the same letter are not significantly different (*p* < 0.05) among treatments. EB, ethidium bromide; AO, acridine orange; GS, germination speed.

As for GR, both AO and EB profoundly affected seed GS. While low concentrations of AO and EB increased GS, high concentrations delayed seed germination and reduced GS (Fig. 2). For example, treatment with 0.5 mg mL^−1^ AO increased the GS of white seeds by 25% compared to the control, whereas treatment with 5 mg mL^−1^ decreased GS by 75% (Fig. 2A; *p* < 0.05). Treatment with 50 μg mL^−1^ EB increased the GSs of white, yellow, red, and purple seeds by approximately 33%, 33%, 6%, and 0% compared to the control, respectively (Fig. 2B; *p* < 0.05).

### Effects of fluridone and H_2_O_2_ on GR

**Figure 3:**
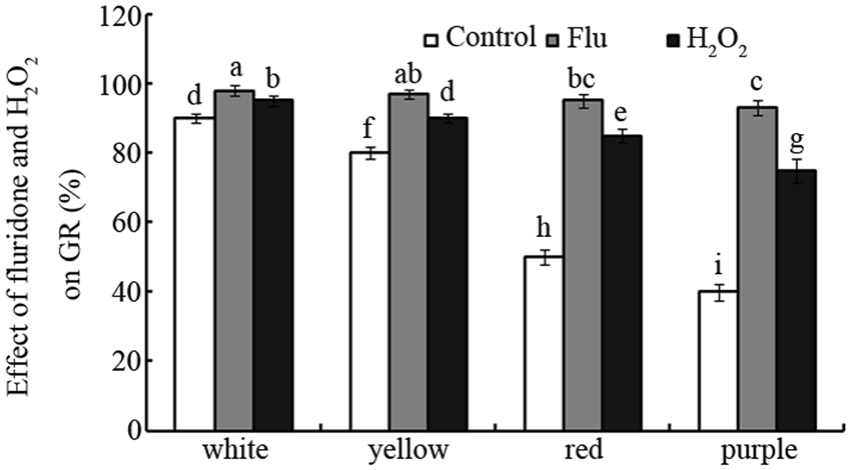
Effects of fluridone and H_2_O_2_ on GR Effects of fluridone and H_2_O_2_ on seed GRs of maize. Bars represent standard deviations of means (*n* = 5), and means followed by the same letter are not significantly different (*p *< 0.05) among treatments. Flu, fluridone; H_2_O_2_, hydrogen peroxide; GR, germination rate.

The effects of fluridone, a specific inhibitor of abscisic acid (ABA) biosynthesis, and H_2_O_2_ on GR were investigated for different-coloured maize seeds (Fig. 3). As shown in Fig. 3, fluridone treatment significantly promoted the dormancy release and increased seed germination, especially for dark-coloured seeds. For example, fluridone increased the final GR by approximately 9%, 21%, 90% and 132% for white, yellow, red and purple seeds, relative to the control group, respectively (Fig. 3; *p* < 0.05). A similar promoting effect was observed following H_2_O_2_ treatment. For example, H_2_O_2_ treatment increased the final GR by approximately 6%, 13%, 70% and 88% for white, yellow, red and purple seeds compared with control seeds, respectively (Fig. 3; *p* < 0.05).

### Effects of AO and EB on H_2_O_2_ content

**Figure 4:**
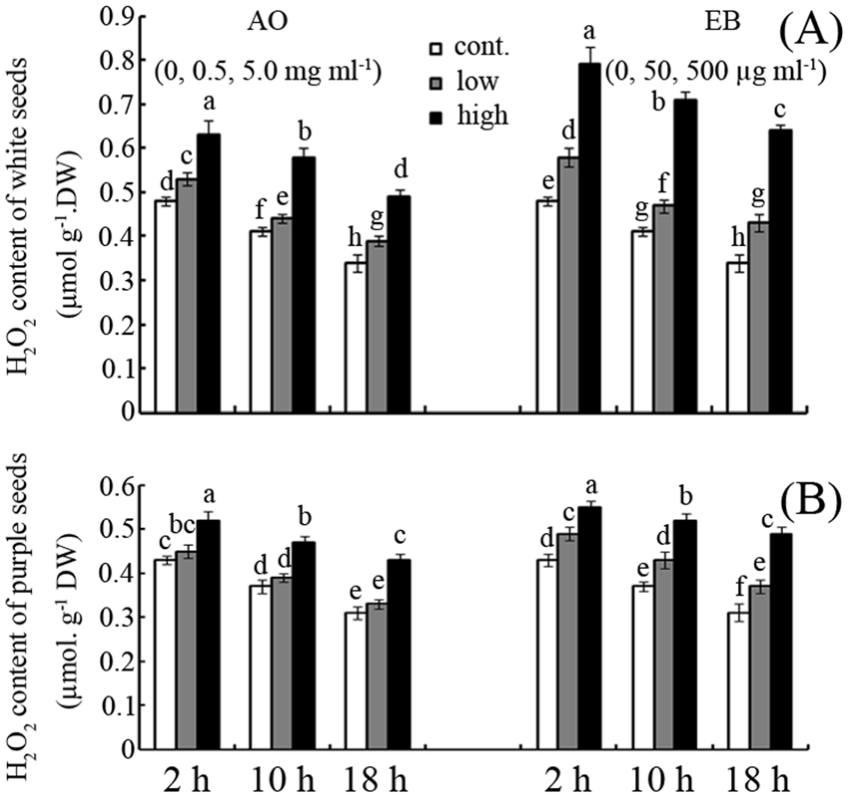
Effects of AO/EB on H_2_O_2_ content Effects of AO and EB on H_2_O_2_ contents of white (**A**) and purple (**B**) seeds. Bars represent standard deviations of means (*n* = 5); means followed by the same letter are not significantly different (*p* < 0.05) among treatments. EB, ethidium bromide; AO, acridine orange; H_2_O_2_, hydrogen peroxide.

The H_2_O_2_ contents of white and purple seeds were determined in this study because these seeds were the most (white) and least (purple) sensitive to the tested mutagens among the seed colours (Fig. 4). Both AO and EB promoted H_2_O_2_ accumulation in a concentration-dependent manner during the first 18 h after seed germination (Fig. 4). For example, treatment with 0.5 and 5 mg mL^−1^ AO increased the H_2_O_2_ content compared to the control by approximately 10% and 31%, 7% and 41%, and 15% and 44% at 2, 10, and 18 h after germination for white seeds, respectively (Fig. 4A; *p* < 0.05). In addition, EB resulted in a greater enhancement of H_2_O_2_ accumulation than AO in white seeds. For example, 0.5 mg mL^−1^ EB increased H_2_O_2_ accumulation by approximately 25%, 22% and 31% compared with 5 mg mL^−1^ AO treatment of white seeds after germination for 2, 10 and 18 hours, respectively (Fig. 4A; *p* < 0.05). The general effects of AO and EB on H_2_O_2_ accumulation in purple seeds were similar to those in white seeds, although the magnitude of the effect was much smaller (Fig. 4B). For example, in purple seeds, treatment with the high concentration of EB (0.5 mg mL^−1^) increased the H_2_O_2_ content by approximately 28%, 41%, and 58% compared to the control at 2, 10, and 18 h after germination, respectively (Fig. 4B; *p* < 0.05).

### Effect of AO and EB on NOX activities

The NOX activity of white seeds were determined in this study because these seeds were the most sensitive to the tested mutagens among the different seed colours tested (Table 1). Both AO and EB promoted NOX activity in a concentration-dependent manner during the first 18 h after seed germination (Table 1). For example, treatment with 0.5 and 5 mg mL^−1^ AO increased NOX activity relative to the control by approximately 15% and 73%, 10% and 87%, and 11% and 70% at 2, 10, and 18 h after germination for white seeds, respectively (Table 1; *p* < 0.05). In addition, EB resulted in a greater enhancement of NOX activity than AO in white seeds. For example, 0.5 mg mL^−1^ EB increased NOX activity by approximately 29%, 34% and 60% compared to the 5 mg mL^−1^ AO treatment for white seeds after germination for 2, 10 and 18 hours, respectively (Table 1; *p* < 0.05).

**Table 1:** Effects of mutagens on NOX activity in different coloured maize seeds. Effects of different concentrations of AO and EB on the NOX activity of white maize seeds. Means followed by the same letter are not significantly different (*p* < 0.05) among different treatments. For each treatment, five replicates were analysed. EB, ethidium bromide; AO, acridine orange; NOX, NADPH oxidase.

NOX activity (U mg^−1^ protein)			
	2 h	10 h	18 h
Control	8.5 ± 0.4^a^	6.3 ± 0.2^a^	4.8 ± 0.3^a^
AO (0.5 mg ml^−1^)	9.8 ± 0.3^b^	6.9 ± 0.3^b^	5.3 ± 0.5^a^
AO (5.0 mg ml^−1^)	14.7 ± 0.5^d^	11.8 ± 0.4^d^	8.2 ± 0.5^c^
EB (50 μg ml^−1^)	11.9 ± 0.4^c^	8.1 ± 0.4^c^	6.2 ± 0.3^b^
EB (500 μg ml^−1^)	18.9 ± 0.7^e^	15.8 ± 0.5^e^	13.2 ± 0.7^d^

### Effects of DMTU and DPI on seed germination

**Figure 5:**
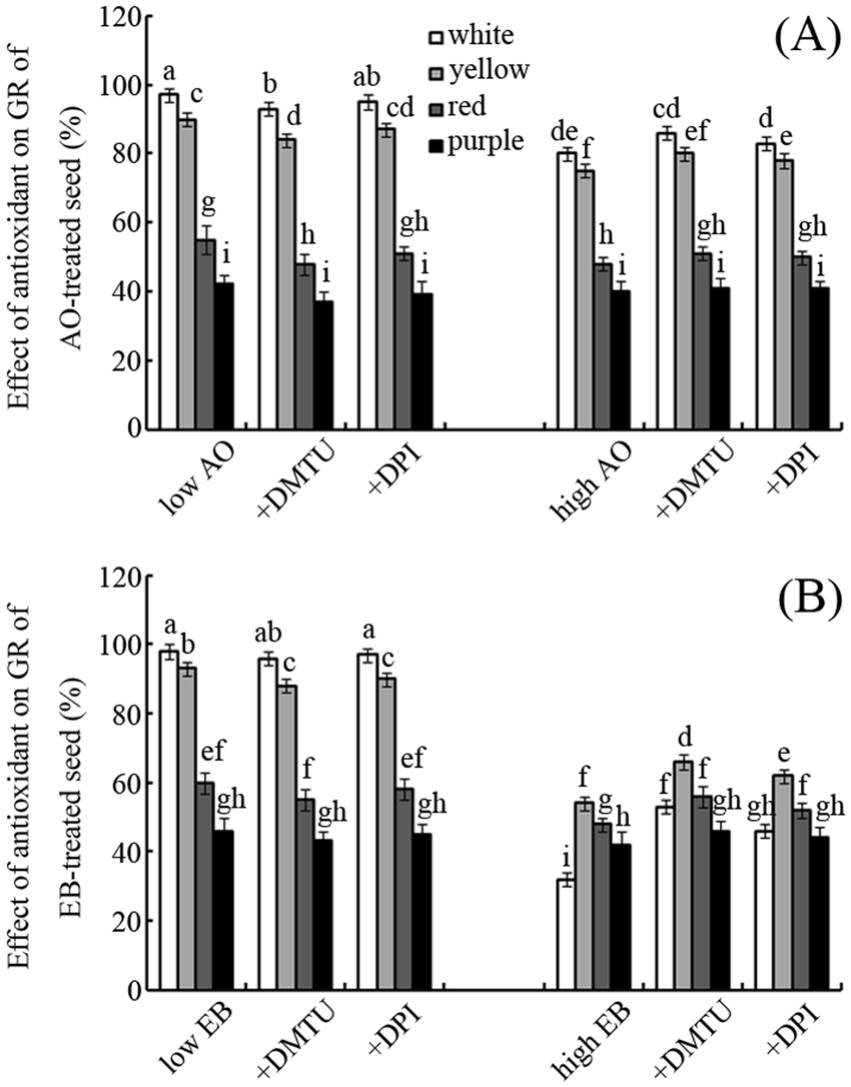
Effects of DMTU and DPI on GR Effects of DMTU and DPI on the GRs of maize seeds treated with low (left) or high (right) concentrations of AO (**A**) or EB (**B**). Bars represent standard deviations of means (*n* = 5); means followed by the same letter are not significantly different (*p* < 0.05) among treatments. Low (0.5 mg ml^−1^ for AO, 50 μg ml^−1^ for EB) or high (5 mg ml^−1^ for AO, 500 μg ml^−1^ for EB) concentrations of AO/EB represent controls. DMTU, dimethyl thiourea; DPI, diphenylene iodonium; AO, acridine orange; EB, ethidium bromide; GR, germination rate.

As shown in Fig. 5, treatment with DMTU could reverse the effects of AO and EB on seed germination, especially for white and yellow seeds. For example, DMTU treatment significantly increased the GRs of white, yellow, red, and purple seeds treated with a high concentration of EB (0.5 mg mL^−1^), by approximately 66%, 22%, 17%, and 10% compared with seeds treated with EB alone (Fig. 5B; *p* < 0.05); treatment with DPI had a similar effect, with corresponding increases in GR of 44%, 15%, 8%, and 5%, respectively (Fig. 5B; *p* < 0.05).

### Effects of H_2_O_2_ and ATZ on seed germination

**Figure 6:**
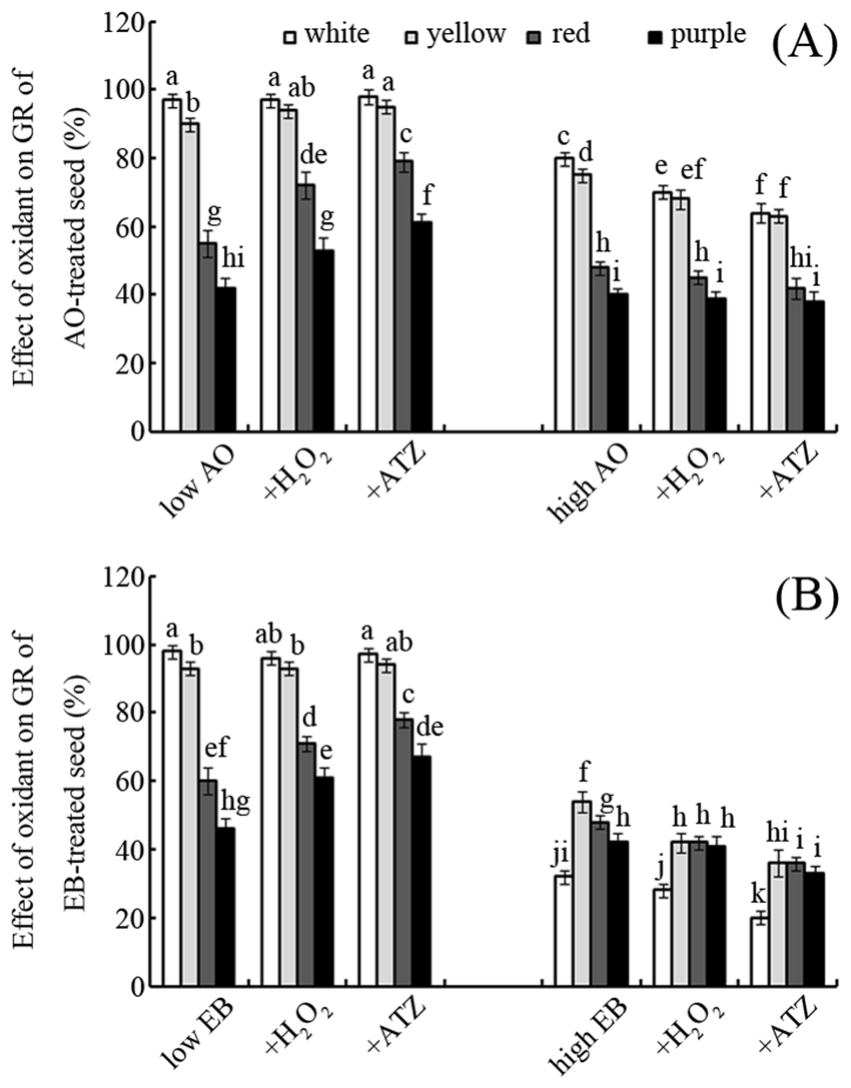
Effects of oxidants on GR Effects of oxidants (H_2_O_2_ and ATZ, 10 mM) on the GRs of maize seeds treated with low (left) or high (right) concentrations of AO (**A**) and EB (**B**). Bars represent standard deviations of means (*n* = 5); means followed by the same letter are not significantly different (*p* < 0.05) among treatments. Low (0.5 mg ml^−1^ for AO, 50 μg ml^−1^ for EB) or high (5 mg ml^−1^ for AO, 500 μg ml^−1^ for EB) concentrations of AO/EB represent controls. H_2_O_2_, hydrogen peroxide; ATZ, aminotriazole; AO, acridine orange; EB, ethidium bromide; GR, germination rate.

As shown in Fig. 6, the effects of AO and EB on seed germination were enhanced by H_2_O_2_, especially for red and purple seeds. Compared with seeds treatment with a high concentrations of AO, treatment with H_2_O_2_ significantly decreased the GRs of white, yellow, red, and purple seeds treated with a high concentration of AO (5 mg mL^−1^), by approximately 13%, 9%, 6%, and 3%, respectively (Fig. 6A; *p* < 0.05). ATZ treatment had a similar effect, producing reductions in the GRs of white, yellow, red, and purple seeds treated by the high concentration of EB (50 μg mL^−1^) of approximately 37%, 33%, 25%, and 21%, respectively (Fig. 6B; *p* < 0.05).

### Effects of AO and EB on seedling growth

**Figure 7:**
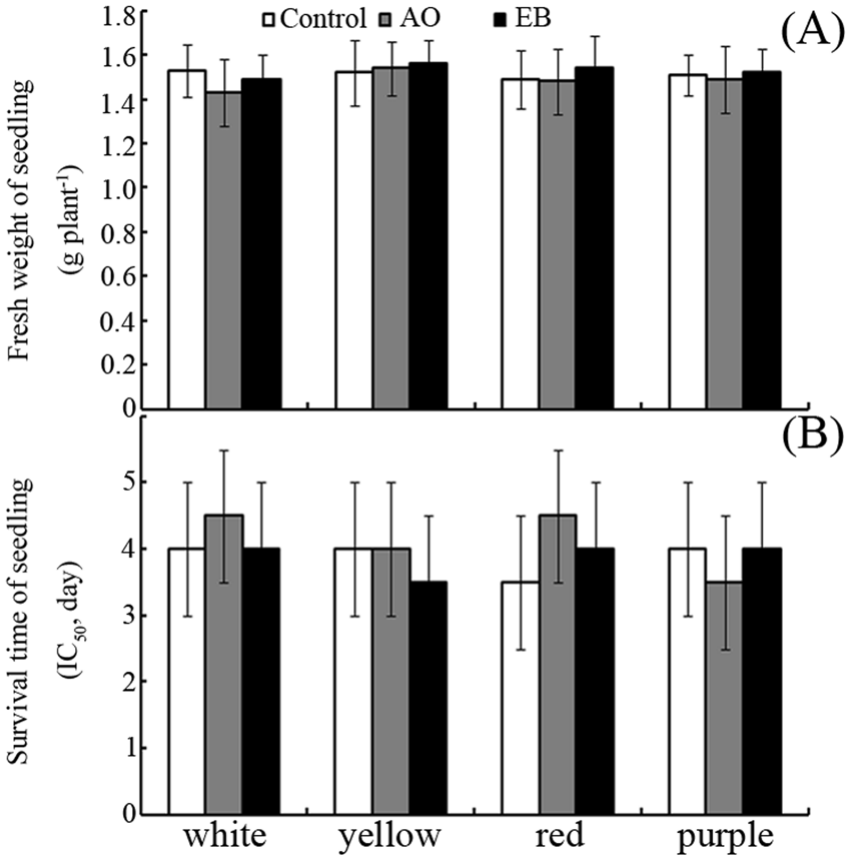
Seedling growth and drought tolerance. (**A**) Fresh weights and (**B**) drought tolerance (evaluated with IC_50_) of maize seedlings grown from mutagen-treated seeds. Bars represent standard deviations of means (*n* = 5); means followed by the same letter are not significantly different (*p* < 0.05) among treatments. AO, acridine orange; EB, ethidium bromide.

As shown in Fig. 7, no significant differences in seedling growth or drought tolerance were observed between seedlings of mutagen-treated seeds, and seedlings of control seeds.

## Discussion

In this study, the effects of two mutagens (EB and AO) on the germination behaviour of different-coloured maize seeds were evaluated (Figs 1 and 2). The results indicate that high mutagen concentrations inhibit seed germination, while low concentrations promote germination (Figs 1 and 2). The “eustress” concept can be used to explain this interesting phenomenon^24^. In contrast with the harmful effects of distress, eustress can enhance functions during the seed life cycle^24^. However, what are the possible mechanisms underlying it?

Compared with the light-coloured seeds, the dark-coloured seeds exhibited lower GR and GS under favourable conditions (Figs 1 and 2). Whether the dark-coloured seeds have a low viability? ABA and antioxidant are known to arrest seed germination^13,25^. However, H_2_O_2_ can degrade ABA, oxidize antioxidant and promote release from seed dormancy^26^. Herein, both fluridone (a specific inhibitor of ABA)^27^ and H_2_O_2_ significantly increased GR, especially for dark-coloured seeds (Fig. 3). This suggests that dark-coloured maize seeds had a high viability (~95%) as well as light-coloured seeds. As for GR, these different-coloured maize seeds exhibited similar sensitivity to fluridone (93~98%) but not to H_2_O_2_ (75~95%) (Fig. 3). It is in accordant with their antioxidant capacity difference among these maize varieties^23^. This suggests that the antioxidant capacity affected their H_2_O_2_ sensitivity for these different-coloured maize seeds. However, it is not known whether these two different mutagens exhibit similar roles to H_2_O_2_ in regulating maize seed germination.

Xenobiotics are known to induce ROS production in plants^28,29^. For example, β-aminobutyric acid promotes ROS production during grapevine-triggered immunity^29^. EB and AO can be considered as xenobiotics in maize plants; thus, EB and AO might induce ROS production in germinating seeds.

As shown in Fig. 4, both EB and AO induced H_2_O_2_ production during maize seed germination in a concentration-dependent manner. The “oxidative window for seed germination” theory proposed by Bailly *et al*. suggests that only favourable amounts of ROS can promote seed germination^13^. In this study, a low concentration of EB or AO produced a favourable amount of ROS, thereby enhancing seed germination. In contrast, a high concentration of EB or AO induced ROS over-production and inhibited seed germination (Figs 1 and 4).

To test this theory, DMTU, a specific ROS scavenger^30^, was applied to mutagen-treated seeds (Fig. 5). Treatment with DMTU partly reversed the inhibitory effects of high concentrations of EB and AO on seed germination (Fig. 5). Accordingly, DMTU can also reduce seed GR under low concentrations of EB or AO availability (Fig. 5). These results suggest that the effects of EB and AO on seed germination can be attributed to the induction of ROS. The possible roles of NADPH oxidase (NOX), an important ROS-produced enzyme^31^, were also investigated. The addition of DPI, a specific inhibitor of NOX^31^, had similar effects on seed germination as the addition of DMTU (Fig. 5). This indicates that NOX-mediated ROS play important roles in the regulation of seed germination by EB and AO. To partially test this hypothesis, the effects of AO and EB on NOX activity in seeds were examined (Table 1). The results showed that these two mutagens, especially EB, significantly enhanced NOX activity in germinating seeds (Table 1). NOX is a key ROS-producing enzyme during seed germination^31^. Interestingly, high NOX activity coupled with high ROS accumulation can be simultaneously monitored in EB- and AO-treated seeds (Table 1; Fig. 4). The results suggest that AO and EB mediate ROS production partly via NOX activation during seed germination. Consistently, reports showed that NOX can sense environmental stimuli and regulate responses in plant cells^32^. Herein, the promotion or inhibition of maize seed germination appeared to be regulated by ROS via NOX, which was activated by both AO and EB.

In this study, the tested mutagens had less of an effect on the germination of dark-coloured seeds than light-coloured seeds (Figs 1 and 2). Our previously published data showed that the antioxidant capacities of variegated maize seeds increase in the following order: white < yellow < red < purple^23^. The results of this study indicate that different coloured maize seeds exhibit differences in sensitivity to AO and EB, with sensitivity decreasing in the order white > yellow > red > purple (Fig. 1). In other words, these mutagens are less toxic to dark-coloured seeds than light-coloured seeds. Thus, it seems that seeds with stronger antioxidant capacity can better defend against the toxicity of these mutagens. H_2_O_2_ and ATZ, a specific inhibitor of catalase^33^, were used to test this hypothesis. Both H_2_O_2_ and ATZ can reduce the antioxidant capacities of seeds (Fig. 6). Interestingly, the oxidant-treated seeds exhibited enhanced sensitivity to EB and AO compared to the control (Fig. 6). This indicates that the toxicity of EB and AO to germination depends partly on the seed antioxidant capacity.

The effects of treating seeds with EB and AO on subsequent seedling growth under favourable or abiotic stress conditions were also examined. No significant differences in fresh weight were observed between the seedlings of mutagen-treated seeds and those of control seeds (Fig. 7A). The mutagen-treated and control seeds also produced seedlings with similar drought stress tolerance (Fig. 7B). In addition, this finding begs the question: why does treating seeds with nucleic acid-binding mutagens not affect subsequent seedling growth and development?

**Figure 8:**
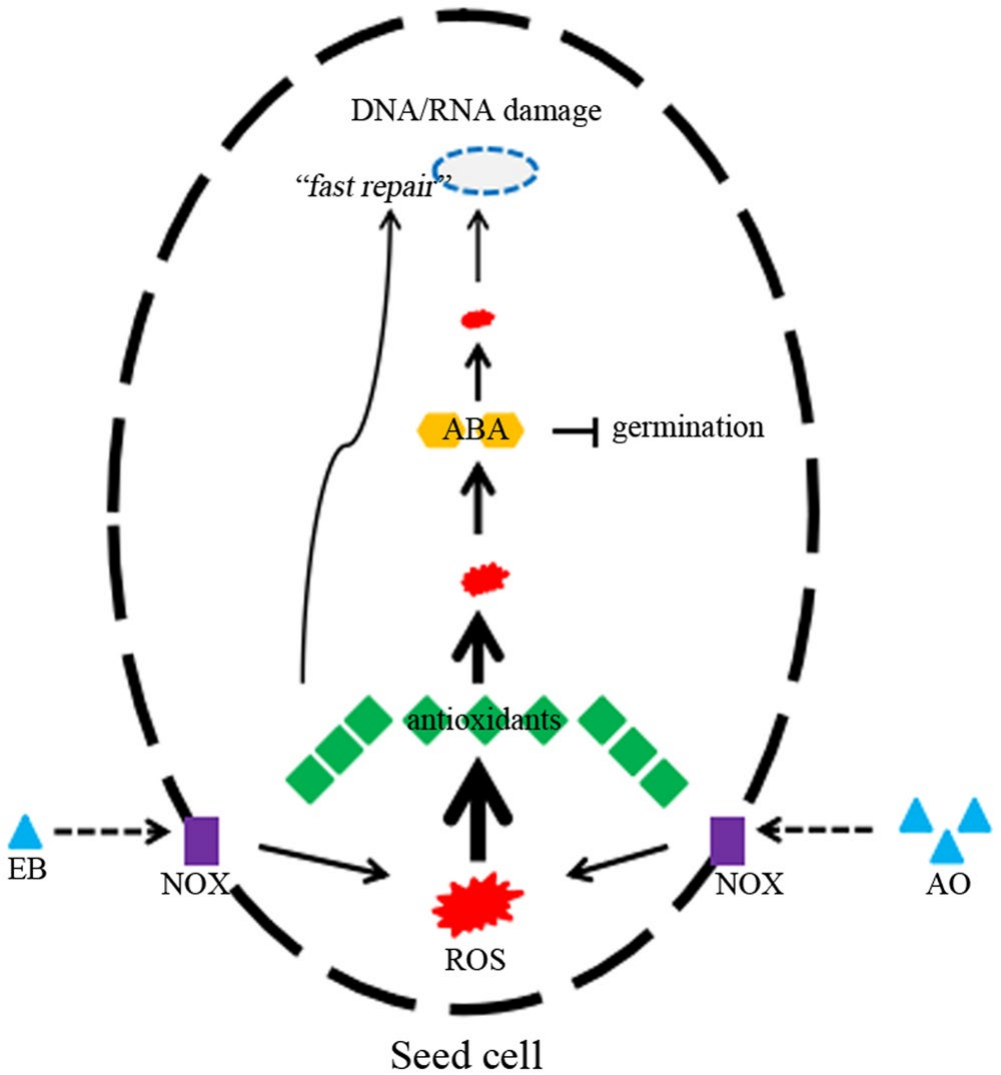
Model of the germination responses of different coloured maize seeds. The hypothetical model, based on ROS scavenging capacity, illustrates the germination responses of different coloured maize seeds following exposure to mutagens. NOX, NADPH oxidase; AO, acridine orange; EB, ethidium bromide; ABA, abscisic acid; ROS, reactive oxygen species.

One plausible answer to this question relates to rapid DNA repair during seed germination. The ‘Fast repair’ theory assumes that antioxidants (such as phenolics) can effectively and instantaneously repair DNA damage^34–36^, which can be induced by free radicals and ROS. Herein, dark-coloured seeds exhibited less phytotoxicity to EB, which could, at least in part, be attributed to fast repair following greater accumulation of antioxidants^23^. Indeed, a number of reports support this conjecture^37,38^. For example, heavy metals (such as Cd, Cu and Pb) can induce genotoxicity, but this can be impeded by antioxidants such as vitamins A, C and E in erythrocytes of Nile tilapia^37^. However, more experimental results are required to fully test this hypothesis. In addition, another possibility is the concentrations of mutagens used in this study are not high enough to impact on seedling development. To illustrate our findings and this interesting phenomenon, a hypothetical model based on ROS scavenging capacity and fast repair theory has been proposed (Fig. 8). In this model, most ROS, produced by mutagen-activated NADPH oxidase, is removed by endogenous antioxidants and ABA, to varying extent. However, remaining ROS can damage nucleic acids, but this can be minimised by fast repair via antioxidants such as polyphenols.

In addition to binding with nucleic acids, AO can also can lead to both deletion- and addition-type frameshift mutations, unlike EB^6^. However, in this study, EB (0.5 mg mL^−1^) had a greater inhibition effect on seed germination than AO (5 mg mL^−1^; Figs 1 and 2). This phenomenon might be attributed to EB’s stronger interference of mitochondrial activities or greater induction of ROS compared to AO (Fig. 4)^19,39^.

While EB (50 μg mL^−1^) was able to significantly promote seed germination, the concentrations used were high compared to those applied typically in laboratories (0.5 μg mL^−1^), and seeds would be unlikely to encounter such high concentrations in the environment. Despite this, our findings expand our understanding of germination improvement by mutagens from a ROS perspective. However, many problems remain, not least the fact that mutagens may share similar functions with EB and AO.

The main conclusions drawn from this study are summarized as follows. Low concentrations of EB and AO improve seed germination, while high concentrations of EB and AO inhibit germination to a degree that depends on seed antioxidant capacity. EB exhibits greater toxicity towards seeds than AO, and the effects of EB and AO on seed germination can be attributed to the induction of ROS. Finally, NOX-mediated ROS are involved in the mechanism by which EB and AO affect seed germination. The treatment of seeds with EB and AO did not affect the growth and drought tolerance of the resultant seedlings, despite the fact that seedling is the most fragile growth stage of the plant lifespan. This indicates that maize plants have greater resistance or adaptation ability to hazardous EB and AO compared to other organisms such as animals and microbes. One plausible explanation for this phenomenon is the greater ROS scavenging capacity of plants compared with other organisms.

## Materials and Methods

### Reagents

All chemical reagents used in this work were of analytical grade. EB and AO were purchased from Sigma-Aldrich (St. Louis, MO, USA). Other reagents were purchased from the Harbin Biochemical Reagent Co., Ltd. (Harbin, China).

### Seed germination and treatment

*Duocainian* maize seeds were sowed in Petri dishes and placed in a seed germinator at 25° ± 1 °C. Germination trials were conducted in 9-cm sterile Petri dishes lined with Whatman No.1 filter papers and moistened with distilled water to ensure adequate seed moisture. All experiments were performed at 25 °C.

This germination experiment can be divided into five groups: EB or AO treatment (group 1), fluridone or H_2_O_2_ treatment (group 2), H_2_O_2_ or 3-amino-1,2,4-triazole (ATZ) treatment (group 3), dimethyl thiourea (DMTU) treatment (group 4), and diphenylene iodonium (DPI) treatment (group 5). All experimental designs were completely randomized with five replicates. In group 1, six concentrations of EB (0, 20, 50, 100, 200, and 500 μg mL^−1^) and AO (0, 0.2, 0.5, 1, 2, and 5 mg mL^−1^) were applied to seeds at 25 °C. As group 2, two concentrations of fluridone (0 and 0.1 mM) and H_2_O_2_ (0 and 10 mM) were applied to seeds at 25 °C. For groups 3, 4 and 5, two H_2_O_2_ or ATZ concentrations (0 and 10 mM), two DMTU concentrations (0 and 10 mM), and two DPI concentrations (0 and 0.1 mM) were sprayed on seeds during the first 24 h after sowing at 25 °C. All assays were replicated at least five times to minimise experimental error, and each replicate germination experiment was carried out on 50 seeds.

### Germination rate assay

Germination was considered to have occurred if the emerged radicle exceeded 1 mm in length. The number of germinated seeds was counted four times per day, and germination rate (GR) was calculated as the percentage of germinated seeds during the first five days after sowing. Germination speed (GS) was determined as the time required to reach 50% of the maximum germination rate.

### H_2_O_2_ extraction and assay

H_2_O_2_ extraction and assay were performed using the methods of Gay *et al*.^40^ with some modification. Treated seeds (water control, EB, and AO) were collected at 2, 10 and 18 h after sowing for H_2_O_2_ assays (white seed germination occurred after 18 h). All germinated seeds were immersed in acetone to terminate germination. The collected seeds were weighed and immediately quenched in liquid N_2_. Samples (~1 g dry weight) were ground to a powder in liquid N_2_ using a mortar and pestle. The ground seeds were then homogenized with 10 mL of 5% trichloroacetic acid (TCA) for H_2_O_2_ extraction. After centrifugation at 10,000 × *g* for 30 min at 25 °C), the supernatants were collected. Xylenol orange reagent was formed by adding 1 mL of assay reagent (25 mM FeSO_4_ and (NH_4_)_2_SO_4_ dissolved in 2.5 M H_2_SO_4_) to 100 mL of 125 μM xylenol orange and 100 mM sorbitol. The supernatant (100 μL) was added to 1 mL of xylenol orange reagent. After 30 min of incubation, the absorbance of the Fe^3+^–xylenol orange complex was recorded at 560 nm.

### NADPH oxidase assay

The NADPH oxidase activity of germinating seeds was determined with a Plant NADPH oxidase ELISA Kit (GENMED SCIENTIFICS INC, USA; GMS50096.3 v.A) in accordance with the instructions of the manufacturer.

### Seedling growth and drought tolerance

After washing 10-day-old maize seedlings with tap water and removing superficial water with bibulous paper, the fresh weights were determined using an electronic balance. To assess the stress tolerance of the plant seedlings, 5-day-old seedlings (from 0.5 mg ml^−1^ EB or 5 mg ml^−1^ AO treatment groups) were grown in pots containing moistening perlite:vermiculite (1:3) under a 16 h:8 h light/dark cycle (200 μmol photon m^−2^ s^−1^) in a greenhouse at a temperature of 22–25 °C and 40–50% relative humidity, and subjected to natural drought (not watered in the following days), and their survival was evaluated based on the half-survival time (IC_50_). Survival time was recorded as the day on which half the plants were considered to be dead.

### Data analysis

All data were analyzed using Duncan’s multiple range test (*p* < 0.05) using SPSS 13.0 software. Five replicates were analyzed for each stress treatment.
